# Exploring the Antidiabetic and Antihypertensive Potential of Peptides Derived from Bitter Melon Seed Hydrolysate

**DOI:** 10.3390/biomedicines12112452

**Published:** 2024-10-25

**Authors:** Wei-Ting Hung, Christoper Caesar Yudho Sutopo, Tunjung Mahatmanto, Mei-Li Wu, Jue-Liang Hsu

**Affiliations:** 1 Department of Food Science, National Pingtung University of Science and Technology, Pingtung 91201, Taiwan; milahung4123@gmail.com; 2 Department and Graduate Institute of Pharmacology, College of Medicine, National Taiwan University, Taipei 10051, Taiwan; 3 Department of Animal Science and Technology, National Taiwan University, Taipei 10672, Taiwan; christopercaesar@gmail.com; 4 Department of Food Science and Biotechnology, Faculty of Agricultural Technology, Universitas Brawijaya, Malang 65145, Indonesia; tjmahatmanto@ub.ac.id; 5 Department of Biological Science and Technology, National Pingtung University of Science and Technology, Pingtung 91201, Taiwan

**Keywords:** *Momordica charantia*, type 2 diabetes, hypertension, active peptide, molecular docking

## Abstract

Background/Objectives: Type 2 diabetes (T2D) has become a critical global health issue, with an increasing prevalence that contributes to significant morbidity and mortality. Inhibiting dipeptidyl peptidase-IV (DPP4) is a promising strategy for managing T2D. This study aimed to explore the DPP4 inhibitory peptide derived from bitter melon seed protein (BMSP) hydrolysate. Methods: Reversed-phase high-performance liquid chromatography (RP-HPLC) was utilized to fractionate the hydrolysate. Peptide in the highest activity fraction was analyzed using liquid chromatography-mass spectrometry (LC-MS/MS). Peptide synthetic was used for further characterizations, such as bioactivity exploration, inhibition mechanism, molecular docking, and peptide stability against in vitro simulated gastrointestinal (SGI) digestion. Results: The BMSP hydrolysate was digested with gastrointestinal proteases (GP) and assessed for DPP4 inhibitory activity, yielding an IC_50_ of 1448 ± 105 μg/mL. Following RP-HPLC fractionation, MPHW (MW4) and VPSGAPF (VF7) were identified from fraction F8 with DPP4 IC_50_ values of 128.0 ± 1.3 µM and 150.6 ± 3.4 µM, respectively. Additionally, MW4 exhibited potential antihypertensive effects through ACE inhibition with an IC_50_ of 172.2 ± 10.6 µM. The inhibitory kinetics and molecular docking simulations indicated that both MW4 and VF7 were competitive inhibitors of DPP4, while MW4 was also a competitive inhibitor of ACE. Importantly, both peptides remained stable during simulated gastrointestinal digestion, suggesting their resistance to human digestive processes and their capacity to maintain biological activity. Conclusions: The findings suggest that BMSP-GP hydrolysate may have potential in terms of the development of health foods or therapeutic agents. However, in vivo studies are also essential for further confirmation of efficacy.

## 1. Introduction

The World Health Organization (WHO) classifies metabolic syndrome as a pathological condition characterized by hyperlipidemia, hypertension, and diabetes [1]. The 10th edition of the International Diabetes Federation (IDF) Diabetes Atlas predicts a significant increase in the global prevalence of diabetes, with the number of cases expected to rise from 537 million in 2021 to 643 million by 2030 [2]. Type 2 diabetes (T2D) accounts for 90% of all diabetes cases, making it the most prevalent form of diabetes. One of the treatments for T2D involves inhibiting the dipeptidyl peptidase-IV (DPP4), which helps lower blood glucose levels while preserving the concentrations of glucagon-like peptide-1 (GLP-1) and glucose-dependent insulinotropic polypeptide (GIP). In 2006, the US Food and Drug Administration (FDA) approved sitagliptin as the first oral antihyperglycemic medication that inhibits DPP4, and it became available in Taiwan in 2009. Currently, the FDA has also approved linagliptin, vildagliptin, and saxagliptin as widely available DPP4 inhibitors [3,4]. However, among the most frequently observed adverse effects of synthetic DPP4 inhibitors are digestive problems such as abdominal pain, nausea, and diarrhea [5,6]. It has been suggested that constituents derived from natural foods or herbs may offer a milder and safer alternative to synthetic forms and be suitable for regulating blood sugar levels [7]. The exploration of DPP4 inhibitory peptides obtained from plant seeds and animal sources has recently expanded, including coix seed prolamin [8], egg yolk protein [9], and *Ruditapes philippinarum* proteins [10]. However, research on DPP4 inhibitory peptides derived from bitter melon seed remains limited.

Hypertension is a common comorbidity in individuals with type 2 diabetes, exacerbating vascular complications and leading to substantial morbidity and mortality. The prevalence of hypertension is notably high among diabetes patients, affecting approximately two out of three individuals [11]. The angiotensin-I-converting enzyme (ACE) is a key component of the blood pressure regulation system in mammals. It converts the inactive decapeptide angiotensin I into the potent vasoconstrictor angiotensin II by cleaving a dipeptide from the C-terminus. Due to its significant role in hypertension, inhibiting ACE is a well established clinical target [12]. The first ACE inhibitory drug was designed and modified based on a sequence from a peptide in the venom of the *Bothrops jararaca* snake. Subsequently, synthetic ACE inhibitors such as captopril, enalapril, and lisinopril have been widely used to manage essential hypertension despite potential adverse effects such as hypotension, hyperkalemia, decreased renal function, and angioedema [13]. ACE inhibitory peptides derived from plant seeds are considered to be milder alternatives to synthetic drugs. Examples include peptides from rapeseed [14], lemon basil seeds [15], sesame seeds [16], and Taiwan red quinoa [17]. Hence, a natural bioactive peptide with both DPP4 and ACE inhibitory activities may be helpful in treating or slowing down complications in diabetes and hypertension.

Whole plants of *Momordica charantia* (bitter melon; BM), including fruit pulp, seed, and leaves, have shown hypoglycemic effects in various animal models [18]. Also, BM has been used in traditional Asian and Indian medicine and has shown clinical antidiabetic activity [18,19]. BM has been revealed as a potential supplementary treatment for metabolic diseases such as diabetes, hypertension, and dyslipidemia [20]. Despite being a byproduct of the food processing industry, BM seeds contain up to 31% protein, making them a potential source of bioactive peptides [21]. Therefore, this study aimed to explore DPP4 inhibitory activity peptides derived from bitter melon seed protein (BMSP) digested by gastrointestinal proteases (GP; combined pepsin, α-chymotrypsin, and trypsin). This study revealed that peptide-derived bitter melon may offer dual benefits, aiding in the management of T2D while serving as a potential antihypertensive therapeutic agent for patients with both diabetes and hypertension.

## 2. Materials and Methods

### 2.1. Materials

Bitter melon seeds were collected in Pingtung County, Taiwan. Trifluoroacetic acid (TFA), recombinant human dipeptidyl peptidase-IV (DPP4), Gly-Pro p-nitroanilide hydrochloride (GP-pNA), p-nitroaniline (pNA), linagliptin, angiotensin-I-converting enzyme (ACE) from rabbit lungs, hippuryl-histidyl-leucine (HHL), hippuric acid (HA), ferulic acid, captopril, pepsin sourced from hog stomach, along with trypsin and α-chymotrypsin obtained from bovine pancreas, were acquired from Sigma–Aldrich Co. (Saint Louis, MO, USA). The following reagents were also used: boric acid, trichloroacetic acid (TCA), sodium dodecyl sulfate (SDS), sodium hydroxide (NaOH), hydrochloric acid (HCl), acetonitrile (ACN), and sodium chloride (NaCl), acetone, dimethylformamide (DMF), diethyl ether, formic acid, and methanol, which were were purchased from J. T. Baker (Phillipsburg, NJ, USA). Wang resin and HBTU were obtained from Creo Salus (Louisville, KY, USA), while Fmoc-amino acids and OxymaPure were sourced from CEM Co. (Matthews, NC, USA). The deionized water was produced using a PURELAB^®^ water purifier (Lane End, High Wycombe, UK). All other chemicals utilized in this research were analytical or HPLC grade.

### 2.2. Bitter Melon Seed Protein (BMSP) Extraction

Concisely, 500 milliliters of 1% SDS solution were combined with 50 g of pulverized bitter melon seed (BMS). To break down the cell membranes of BMS, a digital ultrasonic homogenizer from Branson Ultrasonic (Terra Universal Inc., Fullerton, CA, USA) was set to a 30% duty cycle and operated for 10 min, alternating between 20 s on and off. The supernatant was obtained through a 5 min centrifugation at 8000 rpm. After adding 20% TCA in icy acetone at a 1:1 (*v*/*v*) ratio, the mixture was incubated at −20 °C for 12 h. The lyophilized protein precipitate was washed with water and subsequently stored in a dehumidifying cabinet.

### 2.3. Bitter Melon Seed Protein Gastrointestinal Proteases Hydrolysate Preparation

Bitter melon seed protein (BMSP) was hydrolyzed using gastrointestinal protease (GP; a combination of pepsin, α-chymotrypsin, and trypsin). A 1:50 (*w*/*w*) enzyme-to-protein ratio was employed for the hydrolysis of BMSP. Initially, BMSP was digested by pepsin for 16 h at 37 °C in a 35 mM sodium chloride solution with a pH of 2. The pH was adjusted to 8 by adding 10 N NaOH. α-chymotrypsin and trypsin were subsequently added at 37 °C for another 16 h. After 32 h of enzymatic reaction, hydrolysis was terminated by boiling for 15 min. The BMSP-GP hydrolysate was subjected to centrifugation at 14,000× *g* for 15 min at a temperature of 4 °C. The resulting supernatant was filtered using an Amicon^®^ Ultra 3 kDa molecular weight cut-off (MWCO) ultrafiltration membrane obtained from Merck Millipore (Darmstadt, Germany). This was followed by desalting with a HYPERSEP Retain PEP C_18_ cartridge sourced from Thermo Fisher Scientific (San Jose, CA, USA). The lyophilized hydrolysate, with a molecular weight of less than 3 kDa, was then stored in a dry cabinet.

### 2.4. Determination of DPP4 Inhibitory Activity and IC_50_

The DPP4 inhibitory activity was studied following Hung et al. [22]. All the reagents and samples were dissolved in 0.1 M Tris-HCl buffer, pH 8. First, 25 µL of 1.6 mM GP-pNA was premixed with 25 µL of sample and incubated at 37 °C for 10 min. Then, 50 µL of 0.5 U/µL DPP4 was added. The reaction was set at 37 °C for one hour. 100 µL of 1 M sodium acetate at pH 4 was added to stop the enzymatic reaction. Using a PowerWave XS (Biotek^®^, Winooski, VT, USA) 96-well microplate reader, the absorbance of pNA, which was generated by the DPP4 and GP-pNA reactions, was measured at 405 nm. DPP4 inhibitory activity was examined by the following formula:

(1) $$DPP4\;inhibitory\;activity\left(\%\right)=\left(\frac{pNA(no\;inhibitor)-pNA(inhibitor)}{pNA(no\;inhibitor)}\right)\times 100\%,$$

The absorbances of pNA obtained in the presence and absence of an inhibitor were referred to as pNA (inhibitor) and pNA (no inhibitor), respectively. The DPP4 half-maximal inhibitory concentration (DPP4 IC_50_) was determined by plotting at least five logarithmic concentrations of the inhibitor versus their DPP4 inhibition (%) to construct a nonlinear regression.

### 2.5. Determination of ACE Inhibitory Activity and IC_50_

The ACE inhibitory activity was investigated following Cushman and Cheung [23], with some modifications. First, 30 µL of 2.5 mM HHL was premixed with 10 µL of the sample at 37 °C for 5 min. Then, 20 µL of 0.05 mU/µL ACE was added. The reaction was maintained at 37 °C for one hour. Afterward, 60 µL of 1 N HCl was added to stop the reaction, and 10 µL of 0.2 µg/µL ferulic acid was used as an internal standard. All the reagents and samples were dissolved in pH 8.3 ACE buffer (760 mM boric acid, 300 mM NaCl, and 200 mM NaOH). Using UV-Vis RP-HPLC (Hitachi High-Tech Co., Tokyo, Japan), the area of HA, as the product of ACE and HHL reactions, was measured at 228 nm. An elution with 23% ACN and 0.1% TFA was used at a flow rate of 1 mL/min for 15 min to separate the analytes. ACE inhibitory activity was determined by the following formula:

(2) $$ACE\;inhibitory\;activity\left(\%\right)=\left(\frac{HA(no\;inhibitor)-HA(inhibitor)}{HA(no\;inhibitor)}\right)\times 100\%$$

The areas of HA acquired in the presence and absence of an inhibitor were referred to as HA (inhibitor) and HA (no inhibitor), respectively. The ACE half-maximal inhibitory concentration (ACE IC_50_) was performed by plotting at least five logarithmic concentrations of the inhibitor versus their ACE inhibition (%) to construct a nonlinear regression.

### 2.6. Bioassay-Guided Fractionation of BMSP-GP Hydrolysate

BMSP-GP hydrolysate was separated using reversed-phase high-performance liquid chromatography (RP-HPLC) with a Nucleodur C_18_ semi-preparative column (10 mm × 250 mm × 5 µm, Macherey-Nagel GmbH & Co. KG, Düren, Germany). Solution A (5% ACN containing 0.1% TFA) and solution B (95% ACN containing 0.1% TFA) were used as the mobile phases. Maintaining a flow rate of 4 mL/min and employing UV detection at 214 nm, the elution gradient was set as follows: an isocratic phase of 10% B for the first 5 min, a linear increase from 10% to 35% B over 5 to 55 min, and an isocratic phase of 35% B from 55 to 60 min. The RP-HPLC fractionation resulted in the separation of the BMSP-GP hydrolysate into 18 distinct fractions. Each fraction was freeze-dried and the DPP4 inhibitory activity was assessed for all fractions. Furthermore, peptide sequences from the most active RP-HPLC fraction were identified through a HR-MS/MS analysis combined with peptide de novo sequencing and database searching.

### 2.7. HR-MS/MS Analysis for Peptide Identification Assisted by De Novo Sequencing and Database Search

Peptides in RP-HPLC fraction F8 were analyzed using a Q Exactive Plus Hybrid Quadrupole-Orbitrap mass spectrometer obtained from Thermo Scientific Inc. (Waltham, MA, USA) in conjunction with an UltiMate 3000 RSLCnano system acquired from Dionex (Thermo Scientific Inc., Sunnyvale, CA, USA) and an Acclaim™ PepMap™ C18 column (75 µm × 150 mm, 2 μm, Thermo Scientific Inc., Waltham, MA, USA). The LC system was conducted at a consistent 0.25 μL/min flow rate. Solution A (H_2_O containing 0.1% FA) and solution B (95% ACN containing 0.1% FA) comprised the mobile phases. The elution gradient was programmed as an isocratic gradient of 1% B from 0 to 5.5 min, a linear gradient of 1–30% B from 5.5 to 45 min, a linear gradient of 30–60% B from 45 to 48 min, a linear gradient of 60–80% B from 48 to 50 min, and an isocratic elution of 80% B from 50 to 60 min; additionally, a programmed gradient of 80–1% B was applied from 60 to 65 min, followed by an isocratic elution at 1% B from 65 to 70 min. A data-dependent acquisition method was employed, with the mass range for MS scanning being set between *m/z* 100–1500. MS and MS2 spectra were processed using PEAKS X+ software version 10.6 (Bioinformatics Solution Inc., Waterloo, ON, Canada). The protein database for *Momordica charantia* was retrieved from the NCBI database [24] and imported to PEAKS X+. Concurrently, database matching and peptide de novo sequencing were executed within PEAKS X+ to acquire the peptide sequence.

### 2.8. Synthetic Peptides Preparation

As described in our previous study [25], peptides were synthesized in a peptide synthesizer, with Wang resin serving as the support resin. RP-HPLC was employed to purify the synthetic peptide using parameters similar to those described in the Section 2.6 bioassay-guided RP-HPLC fractionation of BMSP-GP hydrolysate.

### 2.9. Investigation of Peptide Inhibition Mechanism Toward DPP4 and ACE

The inhibition mechanism of peptides on DPP4 and ACE was studied using the inhibitory assays described previously. The peptide inhibition mechanism toward DPP4 was identified by generating a Lineweaver–Burk plot using the production rates of pNA in the presence and absence of the inhibitor at GP-pNA concentrations of 0.8, 1.6, and 3.2 mM. Meanwhile, the HA production rate was determined with and without the inhibitor using HHL concentrations of 0.625, 1.25, and 5 mM to assess the peptide inhibition mechanism against ACE through a Lineweaver–Burk plot.

### 2.10. Molecular Docking Simulation of Peptide Toward DPP4 and ACE

To clarify the molecular-level interaction of peptides with DPP4 and ACE, Discovery Studio 3.0 was employed using the integrated CHARMm (Chemistry at HARvard Macromolecular Mechanics) force field for dynamic energy calculation and molecular docking simulations. Being employed as receptor targets, the crystal structures of human DPP4–diprotin A (PDB Code = 1WCY) [26] and human ACE–lisinopril (PDB Code = 1O86) [27] were acquired from the Protein Data Bank. Peptides were subjected to blind docking with DPP4 utilizing a spherical binding domain (SBD) of 32.7 Å positioned at coordinates x: 107.8, y: 57.5, and z: 42.1. At the same time, blind docking simulations of peptides interacting with ACE were conducted using a 35.5 Å SBD sphere located at x: 39.2, y: 37.7, and z: 50.0. All molecular docking simulations were performed in triplicate. The CDOCKER energy score was applied to ascertain the optimal conformation.

### 2.11. Stability of MW4 and VF7 Against Simulated Gastrointestinal (SGI) Digestion

The peptide stability against SGI digestion was investigated by following the research of Gu and Wu [28], with some modifications. All the SGI enzyme-to-peptide ratios were fixed at 2%. The synthesized peptides MW4 and VF7 were dissolved in 35 mM NaCl buffer (pH 2.0) and digested by pepsin at 37 °C for ninety minutes, respectively. Next, the pH was adjusted to 7–8. Half of each sample was boiled for 15 min to terminate pepsin digestion. The other half of the sample was treated with trypsin and α-chymotrypsin and the reaction was continued at 37 °C for three hours, followed by boiling for 15 min to stop the reaction. An LC-MS analysis was employed to assess the resistance of peptides to SGI digestion.

### 2.12. Statistical Analysis

The results were analyzed in triplicate and presented as the mean ± standard deviation, utilizing GraphPad Prism 9.0 (La Jolla, CA, USA) for analysis. A one-way ANOVA was conducted in SPSS 25.0 (Chicago, IL, USA) for the statistical analyses, which were followed by a Duncan’s multiple range test (*p* < 0.05).

## 3. Results

### 3.1. DPP4 Inhibitory Assay of Bitter Melon Seed Protein (BMSP) Hydrolysate

**Figure 1:**
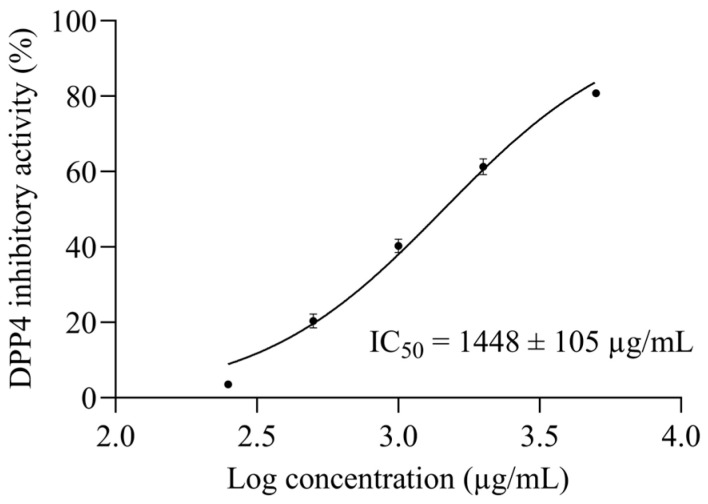
The DPP4 IC_50_ of BMSP-GP.

The DPP4 inhibitory activity of BMSP-GP hydrolysate was investigated. The half-maximal inhibitory concentration of a substance toward a specific biological function is commonly used to measure the efficacy of that substance [29]. Using five logarithmic concentrations of BMSP-GP hydrolysate versus their DPP4 inhibitory activities (%), the half-maximal DPP4 inhibitory concentration (DPP4 IC_50_) was determined to be 1448 ± 105 µg/mL (Figure 1).

### 3.2. Fractionation of BMSP-GP

**Figure 2:**
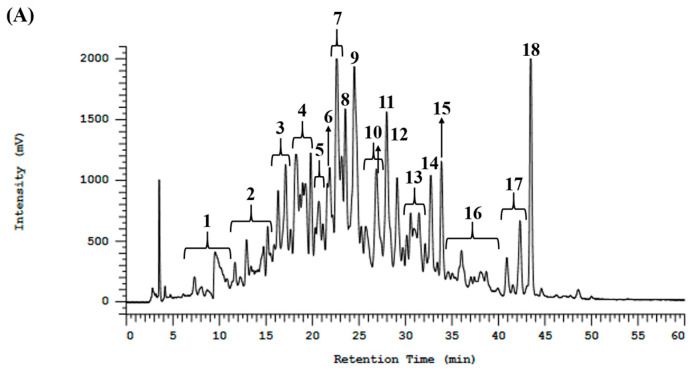

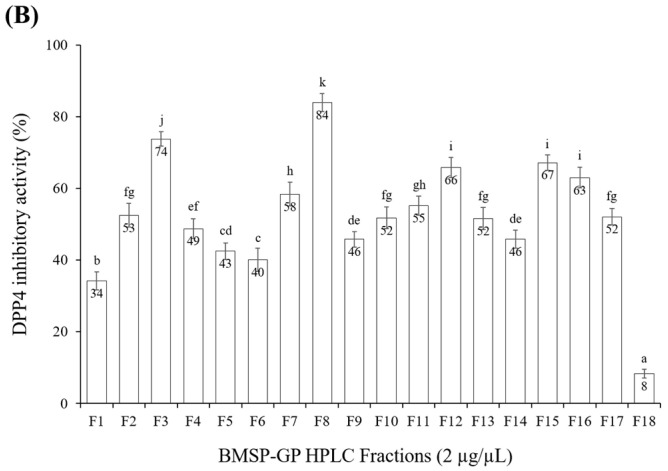
(**A**) RP-HPLC fractionation of BMSP-GP. (**B**) DPP4 inhibitory activity of RP-HPLC fractionation.

Bioassay-guided fractionation is a common strategy for screening and reducing active peptide candidates from peptide mixtures before peptide identification. For decreasing active peptide candidates from protein hydrolysates, reversed-phase (RP) chromatography is widely employed [30]. Therefore, bioassay-guided reversed-phase high-performance liquid chromatography (RP-HPLC) was utilized to fractionate BMSP-GP hydrolysate into eighteen fractions, as shown in Figure 2A. At a concentration of 2 µg/µL, fraction eight (F8) exhibited greater DPP4 inhibitory activity (84 ± 3%) compared to the other fractions (Figure 2B). Peptides contained in F8 were subsequently identified using a high-resolution mass spectrometry (HR-MS/MS) analysis, coupled with database matching and peptide de novo sequencing.

### 3.3. Peptide Sequence Identification

**Figure 3:**
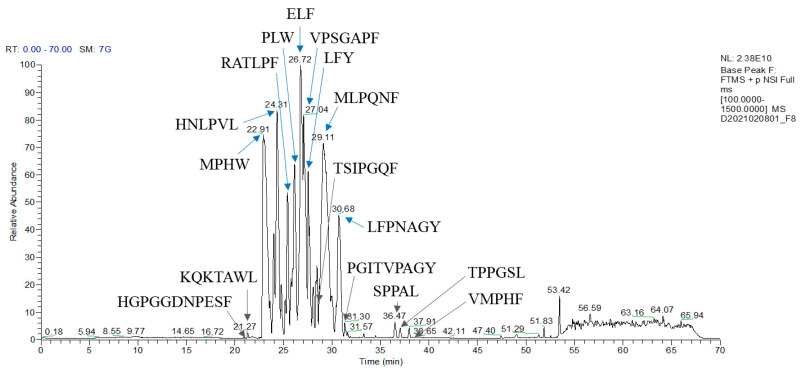
HR-MS/MS chromatogram of F8.

Peptides from F8 were analyzed by HR-MS/MS. The MS and MS/MS raw data were interpreted using PEAKS X+ software version 10.6 to identify the peptide sequences in F8. Sixteen peptides were identified: twelve sequences from de novo peptide sequencing and four sequences from the *Momordica charantia* protein database (Supplementary Table S1). Nine prominent peptide peaks, indicated by blue arrows in Figure 3, were selected. Their MS/MS spectra were manually confirmed (Supplementary Figure S1) and chemically synthesized for further study. Specifically, MPHW (t_R_ 22.91 min), HNLPVL (t_R_ 24.31 min), RATLPF (t_R_ 25.36 min), PLW (t_R_ 26.12 min), ELF (t_R_ 26.72 min), LFY (t_R_ 27.54 min), MLPQNF (t_R_ 29.11 min), and LFPNAGY (t_R_ 30.68 min) were identified using the de novo method. VPSGAPF (t_R_ 27.04 min) was detected in the 187–193 amino acid sequence of the sucrose-binding protein-like isoform X2 (Accession: 1229793389).

### 3.4. The Bioactivity Confirmation of Peptide Candidates from the Fraction F8

**Figure 4:**
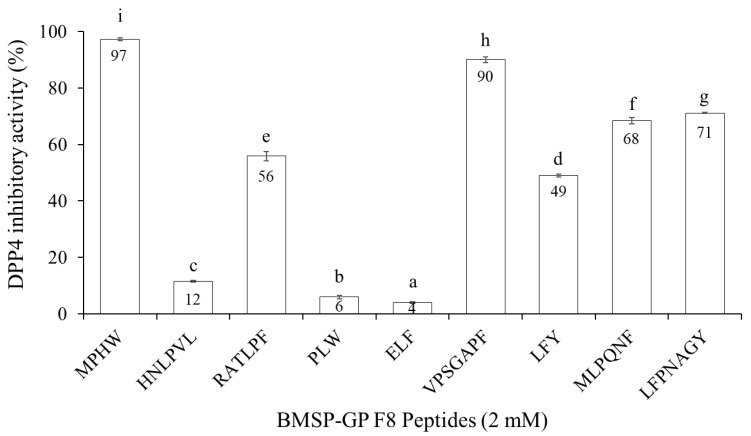
DPP4 inhibitory activity of the peptides derived from F8.

The nine selected peptides were synthesized and their inhibitory activity against DPP4 was confirmed. At the same concentration of 2 mM, MPHW (MW4), HNLPVL (HL6), RATLPF (RF6), PLW (PW3), ELF (EF3), VPSGAPF (VF7), LFY (LY3), MLPQNF (MF6), and LFPNAGY (LY7) exhibited DPP4 inhibitory activity of 97, 12, 56, 6, 4, 90, 49, 68, and 71%, respectively, as shown in Figure 4. Peptides that inhibit DPP4 activity by approximately 50% at a concentration of 2 mM are considered potential DPP4 inhibitory peptide, and their half-maximal inhibitory concentration on DPP4 will be assessed. The half-maximal inhibitory concentration (IC_50_) is a commonly employed method for determining the inhibitory potency of a substance-specific biological or biochemical function. The IC_50_ values of MW4, RF6, VF7, LY3, MF6, and LY7 toward DPP4 were determined to be 128.0 ± 1.3, 1667.0 ± 45.4, 150.6 ± 3.4, 2099.0 ± 55.2, 956.7 ± 39.2, and 913.6 ± 6.7 µM, respectively, as presented in Table 1. Furthermore, as the most potent DPP4 inhibitory peptide derived from BMSP GP hydrolysate, the ACE inhibitory activity of MW4 and VF7 was explored. MW4 not only displayed significant DPP4 inhibitory activity but also showed potent ACE inhibitory activity, with an ACE IC_50_ value of 172 ± 10.6 µM. In contrast, VF7 at a concentration of 200 µM exhibited only 19% ACE inhibitory activity, indicating that VF7 may not be a potential ACE inhibitory peptide. For further characterization of the inhibitory mechanisms, the DPP4 inhibitory mechanisms of MW4 and VF7, as well as the ACE inhibitory mechanism of MW4, will be studied through enzyme kinetic studies.

### 3.5. Inhibitory Mechanism of MW4 Toward ACE and DPP4 Along with VF7 Toward DPP4

**Figure 5:**
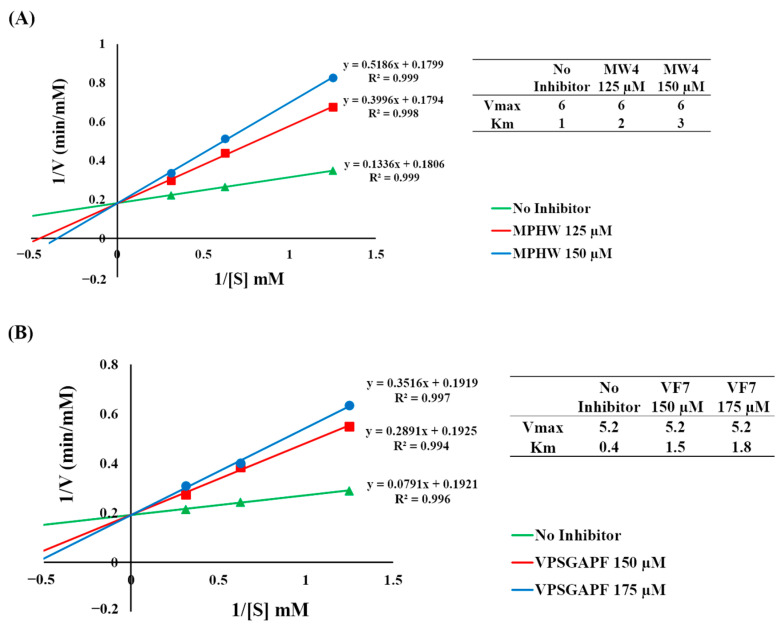
The double reciprocal plot of inhibitor toward DPP4. (**A**) MW4, (**B**) VF7.

The DPP4 inhibition pattern of MW4 and VF7, identified from BMSP-GP hydrolysate, was assessed using a Lineweaver–Burk plot. This analysis involved three different concentrations of substrate (GP-pNA) and was conducted both with and without the addition of an inhibitor. The Lineweaver–Burk plots of MW4 and VF7 (Figure 5A,B) revealed that these peptides inhibited DPP4 in the competitive mechanism, respectively. The Vmax values were stable in the absence or presence of the inhibitors (MW4 and VF7), which indicated that MW4 and VF7 could compete and prevent the DPP4 substrate from binding with the catalytic sites of DPP4.

**Figure 6:**
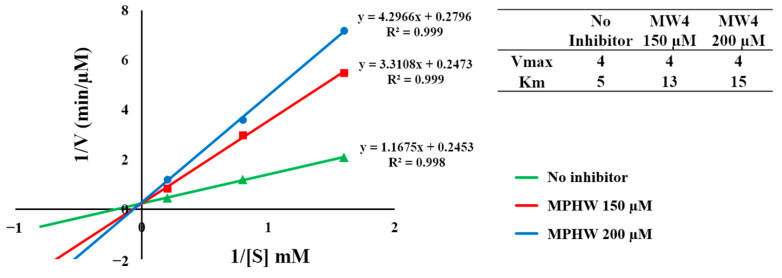
The double reciprocal plot of MW4 toward ACE.

Meanwhile, the ACE inhibition pattern of MW4 was determined using three different concentrations of the substrate (HHL) and was performed both with and without the inclusion of the inhibitor (MW4). The Lineweaver–Burk plot of MW4 (Figure 6) suggested that MW4 inhibited ACE as a competitive inhibitor. The Vmax values were constant in the absence or presence of the inhibitor (MW4). This suggested that the MW4 may be competitive and prevent the substrate from binding with the active sites of ACE.

### 3.6. Intermolecular Interaction Study of MW4 and VF7 Toward DPP4 and ACE Using Molecular Docking Simulation

**Figure 7:**
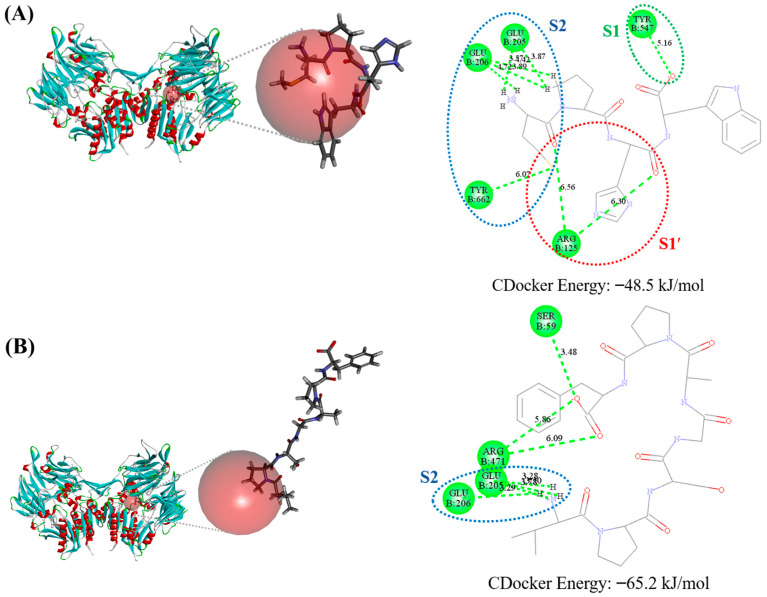
Molecular docking of (**A**) MW4 and (**B**) VF7 toward DPP4.

The molecular docking simulation of MW4 and VF7 toward DPP4 was performed. The interaction between human DPP4 (PDB code: 1WCY) and DPP4 inhibitory peptides (MW4 and VF7) resulted in CDOCKER energies of −48.5 and −65.2 kJ/mol, respectively (Figure 7). MW4 interacted with several DPP4 amino acids residues, including Arg125, Glu205, Glu206, Tyr47, and Tyr662. Meanwhile, VF7 showed interactions with Ser59, Glu205, Glu206, and Arg471.

**Figure 8:**
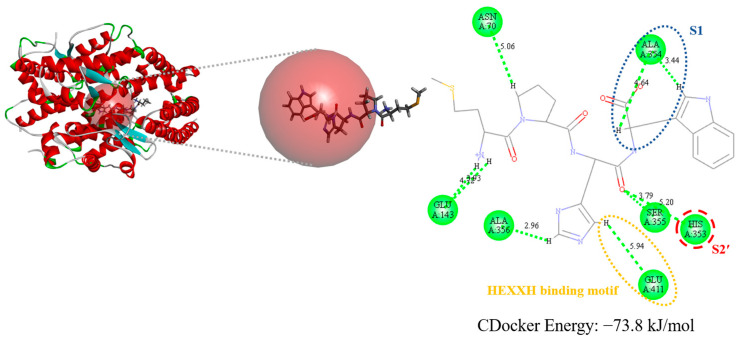
Molecular docking of MW4 toward ACE.

MW4 also exhibited ACE inhibitory activity. Thus, the molecular docking simulation of MW4 toward ACE was also conducted. The docking result of MW4 and human ACE (PDB code: 1O86) resulted in a CDOCKER energy of −73.8 kJ/mol (Figure 8). MW4 formed an intermolecular interaction with ACE amino acid residues, including Asn70, Glu143, His353, Ala354, Ser355, Ala356, and Glu411.

### 3.7. In Vitro Simulated Gastrointestinal (SGI) Digestion Stability of MW4 and VF7

To simulate the stability of MW4 and VF7 during human gastrointestinal digestion, the peptides were incubated with pepsin for ninety minutes, followed by digestion with α-chymotrypsin and trypsin for three hours. Liquid chromatography-tandem mass spectrometry (LC-MS) was employed to observe the stability of MW4 and VF7. As shown in Supplementary Figures S2 and S3, the LC-MS analysis suggested that neither MW4 nor VF7 was hydrolyzed during the simulation of gastrointestinal digestion, as indicated by unaltered *m*/*z* and retention times compared to the control (no digestion).

## 4. Discussion

Diabetes, which includes type 1, type 2, and gestational diabetes, is a global chronic metabolic disorder that affects people in nearly every country, with type 2 diabetes (T2D) being the most prevalent, accounting for about 90% of cases [31]. A direct approach to treating T2D involves prolonging the action of incretin hormones (GLP-1 and GIP), which regulate insulin secretion, by inhibiting DPP-4. Thus, identifying DPP-4 inhibitors is crucial for developing effective antidiabetic agents. Although synthetic inhibitors like vildagliptin, sitagliptin, and saxagliptin are commonly used, their frequent adverse side effects have spurred a growing interest in natural-peptide-product-based therapies. As shown in Figure 1, the BMSP-GP hydrolysate exhibited more potent DPP4 inhibitory activity than *Momordica cochinchinensis* Spreng. seed GP hydrolysate (IC_50_ 1587 µg/mL) [32], as well as hemp, peas, brown rice, and soy proteins digested by simulated gastrointestinal digestion (IC_50_ 1850–4500 µg/mL) [33]. It was comparable to Inca rice (*Chenopodium quinoa* Willd.) bromelain (IC_50_ 900–1120 µg/mL), α-chymotrypsin (IC_50_ 720–980 µg/mL), and pronase E (IC_50_ 830–1020 µg/mL) hydrolysates [34]. Thus, BMSP-GP might contain potential DPP4 inhibitory peptides that are beneficial for the management of T2D.

To screen for DPP4 inhibitory peptides, BMSP-GP hydrolysate was fractionated into 18 fractions using RP-HPLC. Among these, fraction F8 demonstrated significant DPP4 inhibitory activity. The peptides in fraction F8 were analyzed using HR-MS/MS in conjunction with de novo sequencing and database searching. Nine peptides were selected and synthesized for further bioactivity confirmation. Among these nine peptides, MW4 exhibited the highest DPP4 inhibitory activity, with an IC_50_ value of 128.0 ± 1.3 µM. The heptapeptide VF7 also displayed high DPP4 inhibitory activity, with an IC_50_ value of 150.6 ± 3.4 µM, compared to the remaining peptides. The other peptides LY7, MF6, RF6, and LY3, showed considerable IC_50_ values of 913.6 ± 6.7, 956.7 ± 39.2, 1667.0 ± 45.4, and 2099.0 ± 55.2 µM, respectively, as shown in Table 1. DPP4 inhibitory peptides containing proline or alanine at the N-termini penultimate, such as APLVSW (IC_50_ 145 µM) derived from bitter gourd (*Momordica charantia*) seeds GP hydrolysate [22], VPGLAL, LPSW, and LPLF (IC_50_ 270–464 µM) derived from soft-shelled turtle (*Pelodiscus sinensis*) egg yolk GP hydrolysate [35], and PAGPF (IC_50_ 136 µM) derived from rapeseed alcalase and trypsin hydrolysate [36], typically showed favorable DPP4 inhibitory activity [37].

Diabetes and hypertension have been found to coexist more frequently than in isolation. Fifty to seventy percent of people with type 2 diabetes could develop hypertension, and vice versa [11]. Since 1990, controlling diabetes patients’ blood pressure has been linked to reduced cardiovascular disease morbidity and mortality [38]. In diabetic patients with renal illness, drugs that inhibit the renin–angiotensin system maintain the kidneys [39]. Thus, the antihypertensive effect via the ACE inhibitory activity of a DPP4 inhibitory peptide is expected to be beneficial for the management of T2D patients with hypertension, as well as for hypertension patients with T2D. MW4 not only displayed favorable antidiabetic activity via DPP4 inhibitory activity (DPP4 IC_50_ value of 128.0 ± 1.3 µM) but also showed potent antihypertensive activity via ACE inhibitory activity (ACE IC_50_ value 172 ± 10.6 µM). Compared to other active peptides with dual ACE and DPP4 inhibitory activity, MW4 (MPHW) demonstrated comparable inhibitory activities. For instance, LPSW derived from soft-shelled turtle egg yolk GP hydrolysate exhibited dual ACE and DPP4 inhibitory activity with IC_50_ values of 21 and 270 µM, respectively [35,40]. APLVSW derived from bitter gourd seed GP hydrolysate had IC_50_ values of 9.6 and 145.4 µM for ACE and DPP4, respectively [22]. WALPTQSW derived from Inca nut seed GP hydrolysate showed ACE and DPP4 IC_50_ values of 4.7 and 131.7 µM, respectively [41]. It is indicated that dual-function ACE and DPP4 inhibitory peptides usually contain hydrophobic amino acids (A, V, L, I, M, F, W) at the N-terminus, P or A at the penultimate residue position at the N-terminus, and aromatic amino acids (Y, F, W) at the C-terminus.

The inhibitory mechanism study of MW4 and VY7 toward DPP4, as well as MW4 toward ACE, was conducted using the Lineweaver–Burk plot. The Lineweaver–Burk plot for MW4 and VY7 (Figure 5A,B) suggested that these peptides inhibit DPP4 through a competitive mechanism. The Vmax values remained unchanged whether the inhibitors (MW4 and VF7) were present or not, suggesting that MW4 and VY7 compete with the DPP4 substrate for the enzyme’s catalytic sites. These results agreed with the previous study that DPP4 inhibitory peptides with proline or alanine at the N-termini penultimate were competitive inhibitors [42]. Because MW4 is also an ACEI peptide, its inhibitory mechanism toward ACE was assessed. The Lineweaver–Burk plot of MW4 (Figure 6) indicated that MW4 acts as a competitive inhibitor of ACE. This suggests that MW4 competes with the substrate, preventing it from binding to the active sites of ACE. This result aligns with previous studies indicating that ACEI peptides with a C-terminal tryptophan moiety primarily inhibit ACE at its active sites [22,29,41].

Molecular docking simulations between active peptides and target enzymes (DPP4 or ACE) can provide additional insight into their putative intermolecular interactions. According to the interaction between DPP4 and diprotin A (a competitive DPP4 inhibitor), the active site of DPP4 comprises the S1 pocket (Tyr547 and Tyr631), the S1′ pocket (Arg125), and the S2 pocket (Glu205, Glu206, and Tyr662) [26]. As shown in Figure 7, MW4 interacted with the S1 pocket (Tyr547), the S1′ pocket (Arg125), and the S2 pocket (Glu205, Glu206, and Tyr662) residues of the DPP4 active sites. Meanwhile, VF7 interacted with the DPP4 active site residues in the S2 pocket (Glu205 and Glu206), as shown in Figure 8. This putative intermolecular inhibitory mechanism indicates that both peptides can prevent the access of natural substrate, accounting for the DPP4 inhibitory activity of these peptides, which aligns with the inhibitory mechanism study. Additionally, the putative intermolecular inhibitory mechanism of MW4 toward ACE was also studied using molecular docking simulation. Based on the interaction between ACE and lisinopril (an ACE competitive inhibitory) [27], the active site of ACE is composed of four pockets: the HEXXH binding motif (His383, His387, and Glu411), the S1 pocket (Ala354, Glu384, and Tyr523), the S1′ pocket (Glu162), and the S2′ pocket (Gln281, His353, Lys511, His513, and Tyr520) [43]. As shown in Figure 8, MW4 interacted with the HEXXH binding motif (Glu411), S1 pocket (Ala354), and S2′ pocket (His353) of the ACE active site residues. This aligns with the inhibitory mechanism study, confirming that MW4 is an ACE competitive inhibitor. Therefore, both peptides (MW4 and VF7) are competitive DPP4 inhibitors and MW4 is also a competitive ACE inhibitor.

Bioactive peptides are commonly administered orally. Their activity might be altered during gastrointestinal digestion due to the actions of gastrointestinal proteases. Thus, their stability during simulated gastrointestinal (SGI) digestion is an important consideration. MW4 and VF7 were generated by gastrointestinal proteases (GP). In theory, the same proteolytic enzymes will not cleave them. The stability of MW4 and VF7 toward SGI digestion was monitored by LC-MS (Supplementary Figures S2 and S3). As expected, neither MW4 nor VF7 was hydrolyzed during the simulation of gastrointestinal digestion. Low-molecular-weight active peptides (>1 kDa) typically demonstrate greater resistance to gastrointestinal proteases [44]. Previous studies also mentioned that the presence of proline in an active peptide sequence—MW4 (Pro2) and VF7 (Pro2 and Pro6)—might be a imperative feature for their resistance toward simulated gastrointestinal digestion [22,45,46]. Therefore, the resistance of MW4 and VF7 during SGI digestion indicates that these active peptides remain in an active form and may have the potential to exert significant antidiabetic and antihypertensive effects.

## 5. Conclusions

This study presents the antidiabetic potential of BMSP-GP hydrolysate through its DPP4 inhibitory activity. The DPP4 IC_50_ value of BMSP-GP was determined as 1448 ± 105 μg/mL. Using RP-HPLC bioassay-guided fractionations, two potential antidiabetic peptides (MW4 and VF7) were characterized. The DPP4 IC_50_ values of MW4 and VF7 were calculated to be 128.0 ± 1.3 and 150.6 ± 3.4 µM, respectively. MW4 demonstrated a favorable antihypertensive effect via ACE inhibitory activity, with IC_50_ values of 172 ± 10.6 µM. The inhibitory kinetic and molecular docking simulations demonstrated that MW4 and VF7 are competitive inhibitors of DPP4 and that MW4 is also a competitive ACE inhibitor. In addition, MW4 and VF7 are stable during SGI digestion, implying that they may be resistant to hydrolysis by human gastrointestinal digestive enzymes and can maintain their biological activity. Hence, MW4 and VF7 show potential in regard to the development of health foods. It is anticipated that peptide-derived bitter melon will provide dual benefits by aiding in the management of T2D and serving as a potential antihypertensive therapeutic agent, which could be particularly valuable for patients with both diabetes and hypertension.

## Figures and Tables

**Table 1:** DPP4 IC_50_ of the peptides derived from F8.

Identified Peptide	IC_50_ (µM)
MPHW (MW4)	128.0 ± 1.3
RATLPF (RF6)	1667.0 ± 45.4
VPSGAPF (VF7)	150.6 ± 3.4
LFY (LY3)	2099.0 ± 55.2
MLPQNF (MF6)	956.7 ± 39.2
LFPNAGY (LY7)	913.6 ± 6.7

## Data Availability

Data are contained within the article and the Supplementary Materials.

## Supplementary Materials

The following supporting information can be downloaded at: https://www.mdpi.com/article/10.3390/biomedicines12112452/s1, Figure S1: Manually confirmed nine peptides LC-MS/MS spectrum, Figure S2: LC-MS chromatogram and MS spectra of the stability MW4 toward SGI digestion, Figure S3: LC-MS chromatogram and MS spectra of the stability VF7 toward SGI digestion, Table S1: Identified peptides from BMSP-GP F8.

## References

[1] Saklayen M.G. (2018). The global epidemic of the metabolic syndrome. Curr. Hypertens. Rep..

[2] Kumar A., Gangwar R., Ahmad Zargar A., Kumar R., Sharma A. (2024). Prevalence of diabetes in india: A review of idf diabetes atlas 10th edition. Curr. Diabetes Rev..

[3] Karagiannis T., Boura P., Tsapas A. (2014). Safety of dipeptidyl peptidase 4 inhibitors: A perspective review. Ther. Adv. Drug Saf..

[4] Chen Y.-C., Chen T.-H., Sun C.-C., Chen J.-Y., Chang S.-S., Yeung L., Tsai Y.-W. (2020). Dipeptidyl peptidase-4 inhibitors and the risks of autoimmune diseases in type 2 diabetes mellitus patients in taiwan: A nationwide population-based cohort study. Acta Diabetol..

[5] Zhou X.-j., Ding L., Liu J.-X., Su L.-Q., Dong J.-J., Liao L. (2019). Efficacy and short-term side effects of sitagliptin, vildagliptin and saxagliptin in chinese diabetes: A randomized clinical trial. Endocr. Connect..

[6] Choy M., Lam S. (2007). Sitagliptin: A novel drug for the treatment of type 2 diabetes. Cardiol. Rev..

[7] Lacroix I.M., Li-Chan E.C. (2014). Overview of food products and dietary constituents with antidiabetic properties and their putative mechanisms of action: A natural approach to complement pharmacotherapy in the management of diabetes. Mol. Nutr. Food Res..

[8] Zhang S., Li Z.-m., Feng Y., Yu S., Li Z., Zhang D., Wang C. (2023). Dpp-iv inhibitory peptides from coix seed prolamins: Release, identification, and analysis of the interaction between key residues and enzyme domains. J. Agric. Food Chem..

[9] Zambrowicz A., Eckert E., Pokora M., Bobak Ł., Dąbrowska A., Szołtysik M., Trziszka T., Chrzanowska J. (2015). Antioxidant and antidiabetic activities of peptides isolated from a hydrolysate of an egg-yolk protein by-product prepared with a proteinase from asian pumpkin (*Cucurbita ficifolia*). RSC Adv..

[10] Liu R., Zhou L., Zhang Y., Sheng N.-J., Wang Z.-K., Wu T.-Z., Wang X.-Z., Wu H. (2017). Rapid identification of dipeptidyl peptidase-iv (dpp-iv) inhibitory peptides from ruditapes philippinarum hydrolysate. Molecules.

[11] Ferrannini E., Cushman W.C. (2012). Diabetes and hypertension: The bad companions. Lancet.

[12] Guimaraes P.B., Alvarenga E.C., Siqueira P.D., Paredes-Gamero E.J., Sabatini R.A., Morais R.L., Reis R.I., Santos E.L., Teixeira L.G., Casarini D.E. (2011). Angiotensin ii binding to angiotensin i–converting enzyme triggers calcium signaling. Hypertension.

[13] FitzGerald R.J., Murray B.A., Walsh D.J. (2004). Hypotensive peptides from milk proteins. J. Nutr..

[14] Marczak E.D., Usui H., Fujita H., Yang Y., Yokoo M., Lipkowski A.W., Yoshikawa M. (2003). New antihypertensive peptides isolated from rapeseed. Peptides.

[15] Kheeree N., Sangtanoo P., Srimongkol P., Saisavoey T., Reamtong O., Choowongkomon K., Karnchanatat A. (2020). Ace inhibitory peptides derived from de-fatted lemon basil seeds: Optimization, purification, identification, structure–activity relationship and molecular docking analysis. Food Funct..

[16] Aondona M.M., Ikya J.K., Ukeyima M.T., Gborigo T.w.J., Aluko R.E., Girgih A.T. (2021). In vitro antioxidant and antihypertensive properties of sesame seed enzymatic protein hydrolysate and ultrafiltration peptide fractions. J. Food Biochem..

[17] Suleman D.P., Sutopo C.C.Y., Hsu J.-L. (2024). Characterization of novel angiotensin-i converting enzyme inhibitory peptides derived from taiwan red quinoa (*Chenopodium formosanum Koidz*.) seed proteins using two sequential bioassay-guided fractionations. Med. Chem. Res..

[18] Grover J., Yadav S., Vats V. (2002). Medicinal plants of india with anti-diabetic potential. J. Ethnopharmacol..

[19] Fuangchan A., Sonthisombat P., Seubnukarn T., Chanouan R., Chotchaisuwat P., Sirigulsatien V., Ingkaninan K., Plianbangchang P., Haines S.T. (2011). Hypoglycemic effect of bitter melon compared with metformin in newly diagnosed type 2 diabetes patients. J. Ethnopharmacol..

[20] Zeng Y., Guan M., Li C., Xu L., Zheng Z., Li J., Xue Y. (2018). Bitter melon (*Momordica charantia*) attenuates atherosclerosis in apo-e knock-out mice possibly through reducing triglyceride and anti-inflammation. Lipids Health Dis..

[21] Naik M., Natarajan V., Modupalli N., Thangaraj S., Rawson A. (2022). Pulsed ultrasound assisted extraction of protein from defatted bitter melon seeds (*Momardica charantia* L.) meal: Kinetics and quality measurements. LWT—Food Sci. Technol..

[22] Hung W.-T., Sutopo C.C.Y., Wu M.-L., Hsu J.-L. (2023). Discovery and characterization of a dual-function peptide derived from bitter gourd seed protein using two orthogonal bioassay-guided fractionations coupled with in silico analysis. Pharmaceuticals.

[23] Cushman D.W., Cheung H.S. (1971). Spectrophotometric assay and properties of the angiotensin-converting enzyme of rabbit lung. Biochem. Pharmacol..

[24] Protein. Bethesda (md): National Library of Medicine (USA), National Center for Biotechnology Information. https://www.ncbi.nlm.nih.gov/protein/.

[25] Shih Y.-H., Chen F.-A., Wang L.-F., Hsu J.-L. (2019). Discovery and study of novel antihypertensive peptides derived from cassia obtusifolia seeds. J. Agric. Food Chem..

[26] Hiramatsu H., Yamamoto A., Kyono K., Higashiyama Y., Fukushima C., Shima H., Sugiyama S., Inaka K., Shimizu R. (2004). The Crystal Structure of Human Dipeptidyl Peptidase IV (Dppiv) Complex with Diprotin A.

[27] Natesh R., Schwager S.L., Evans H.R., Sturrock E.D., Acharya K.R. (2004). Structural details on the binding of antihypertensive drugs captopril and enalaprilat to human testicular angiotensin i-converting enzyme. Biochemistry.

[28] Gu Y., Wu J. (2016). Bovine lactoferrin-derived ace inhibitory tripeptide lrp also shows antioxidative and anti-inflammatory activities in endothelial cells. J. Funct. Foods.

[29] Sutopo C.C.Y., Aznam N., Arianingrum R., Hsu J.-L. (2023). Screening potential hypertensive peptides using two consecutive bioassay-guided spe fractionations and identification of an ace inhibitory peptide, dhstavw (dw7), derived from pearl garlic protein hydrolysate. Peptides.

[30] Herraiz T. (1997). Sample preparation and reversed phase-high performance liquid chromatography analysis of food-derived peptides. Anal. Chim. Acta.

[31] Saeedi P., Petersohn I., Salpea P., Malanda B., Karuranga S., Unwin N., Colagiuri S., Guariguata L., Motala A.A., Ogurtsova K. (2019). Global and regional diabetes prevalence estimates for 2019 and projections for 2030 and 2045: Results from the international diabetes federation diabetes atlas, 9th edition. Diabetes Res. Clin. Pract..

[32] Ngamsuk S., Hsu J.-L. (2022). Identification of dipeptidyl peptidase iv inhibitory peptides derived from gac seed protein hydrolysate using hydrophilic interaction liquid chromatography and reversed-phase high-performance liquid chromatography. Agric. Nat. Resour..

[33] Nongonierma A.B., FitzGerald R.J. (2015). Investigation of the potential of hemp, pea, rice and soy protein hydrolysates as a source of dipeptidyl peptidase iv (dpp-iv) inhibitory peptides. Food Dig..

[34] Mudgil P., Kilari B.P., Kamal H., Olalere O.A., FitzGerald R.J., Gan C.-Y., Maqsood S. (2020). Multifunctional bioactive peptides derived from quinoa protein hydrolysates: Inhibition of α-glucosidase, dipeptidyl peptidase-iv and angiotensin i converting enzymes. J. Cereal Sci..

[35] Nong N.T.P., Chen Y.-K., Shih W.-L., Hsu J.-L. (2020). Characterization of novel dipeptidyl peptidase-iv inhibitory peptides from soft-shelled turtle yolk hydrolysate using orthogonal bioassay-guided fractionations coupled with in vitro and in silico study. Pharmaceuticals.

[36] Xu F., Yao Y., Xu X., Wang M., Pan M., Ji S., Wu J., Jiang D., Ju X., Wang L. (2019). Identification and quantification of dpp-iv-inhibitory peptides from hydrolyzed-rapeseed-protein-derived napin with analysis of the interactions between key residues and protein domains. J. Agric. Food Chem..

[37] Hatanaka T., Inoue Y., Arima J., Kumagai Y., Usuki H., Kawakami K., Kimura M., Mukaihara T. (2012). Production of dipeptidyl peptidase iv inhibitory peptides from defatted rice bran. Food Chem..

[38] De Boer I.H., Bangalore S., Benetos A., Davis A.M., Michos E.D., Muntner P., Rossing P., Zoungas S., Bakris G. (2017). Diabetes and hypertension: A position statement by the american diabetes association. Diabetes Care.

[39] Jandeleit-Dahm K., Cooper M.E. (2002). Hypertension and diabetes. Curr. Opin. Nephrol. Hypertens..

[40] Nong N.T.P., Sutopo C.C.Y., Hung W.-T., Wu P.-H., Hsu J.-L. (2022). The molecular docking and inhibition kinetics of angiotensin i-converting enzyme inhibitory peptides derived from soft-shelled turtle yolk. Appl. Sci.-Basel.

[41] Sutopo C.C.Y., Hung W.-T., Hsu J.-L. (2024). A simple tandem bioassay-guided scx-rp spe fractionation for efficient active peptide screening from inca nut cake protein hydrolysate. J. Chromatogr. B.

[42] Nongonierma A.B., Paolella S., Mudgil P., Maqsood S., FitzGerald R.J. (2018). Identification of novel dipeptidyl peptidase iv (dpp-iv) inhibitory peptides in camel milk protein hydrolysates. Food Chem..

[43] Andújar-Sánchez M., Cámara-Artigas A., Jara-Pérez V. (2004). A calorimetric study of the binding of lisinopril, enalaprilat and captopril to angiotensin-converting enzyme. Biophys. Chem..

[44] Chen M., Li B. (2012). The effect of molecular weights on the survivability of casein-derived antioxidant peptides after the simulated gastrointestinal digestion. Innov. Food Sci. Emerg. Technol..

[45] Vermeirssen V., Camp J.V., Verstraete W. (2004). Bioavailability of angiotensin i converting enzyme inhibitory peptides. Br. J. Nutr..

[46] Ningrum S., Sutrisno A., Hsu J.-L. (2022). An exploration of angiotensin-converting enzyme (ace) inhibitory peptides derived from gastrointestinal protease hydrolysate of milk using a modified bioassay-guided fractionation approach coupled with in silico analysis. Int. J. Dairy Sci..

