# Supplementary material for: Exploring the Antidiabetic and Antihypertensive Potential of Peptides Derived from Bitter Melon Seed Hydrolysate
Source: Biomedicines. 2024 Oct 25;12(11):2452. doi: 10.3390/biomedicines12112452 (PMC11591893; doi:10.3390/biomedicines12112452)
Supplement: Supplementary file 1 [file biomedicines-12-02452-s001.zip › biomedicines-3238256-supplementary.pdf]

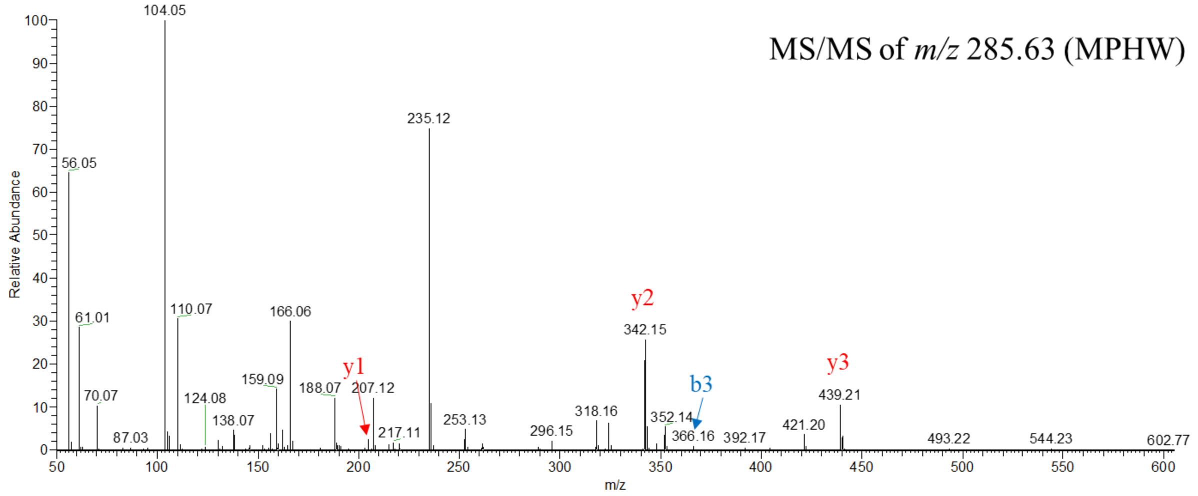

MS/MS of *m/z* 285.63 (MPHW)
Relative Abundance
m/z
104.05
235.12
56.05
61.01
110.07
166.06
y2
342.15
y3
439.21
b3
y1
159.09
188.07
207.12
70.07
124.08
138.07
87.03
217.11
253.13
296.15
318.16
352.14
366.16
392.17
421.20
493.22
544.23
602.77

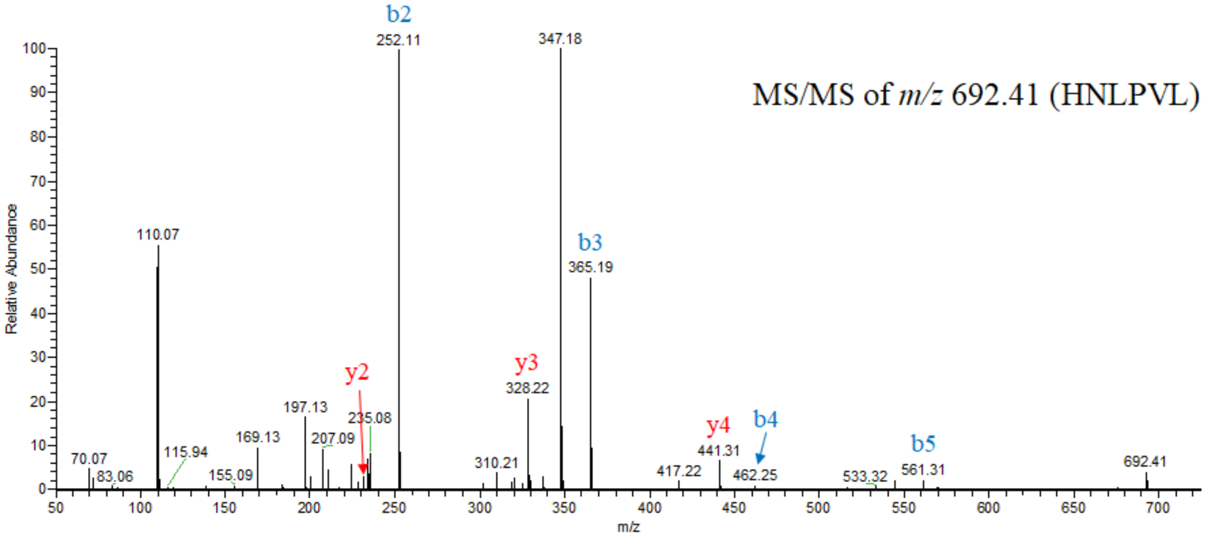

MS/MS of *m/z* 692.41 (HNLPVL)
Relative Abundance
m/z
b2
252.11
347.18
110.07
b3
365.19
y2
y3
328.22
197.13
235.08
169.13
207.09
70.07
83.06
115.94
155.09
310.21
y4
441.31
b4
462.25
417.22
b5
561.31
533.32
692.41

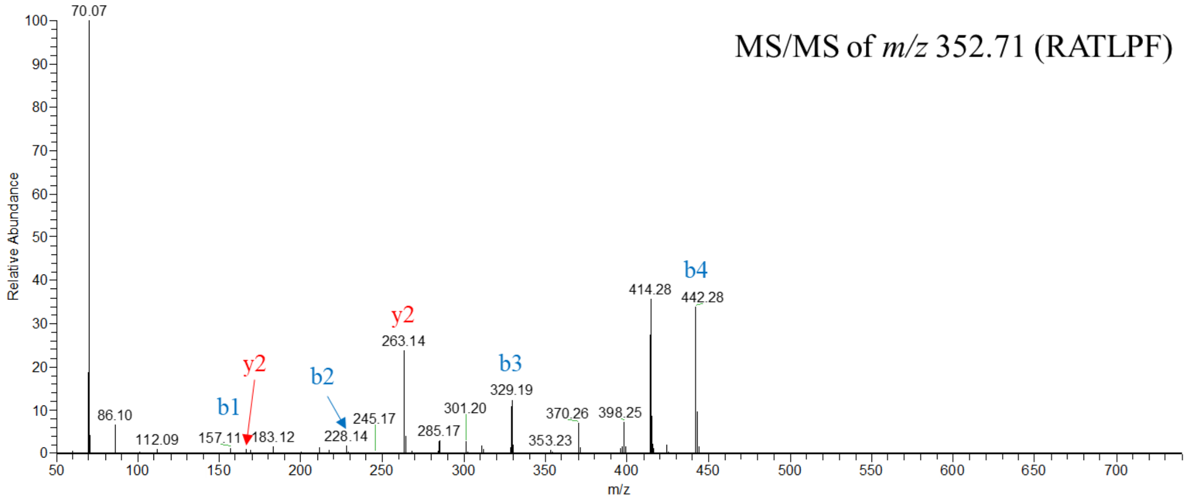

MS/MS of *m/z* 352.71 (RATLPF)
Relative Abundance
m/z
70.07
b4
414.28
442.28
y2
263.14
y2
b2
b3
329.19
b1
86.10
112.09
157.11
183.12
228.14
245.17
285.17
301.20
353.23
370.26
398.25

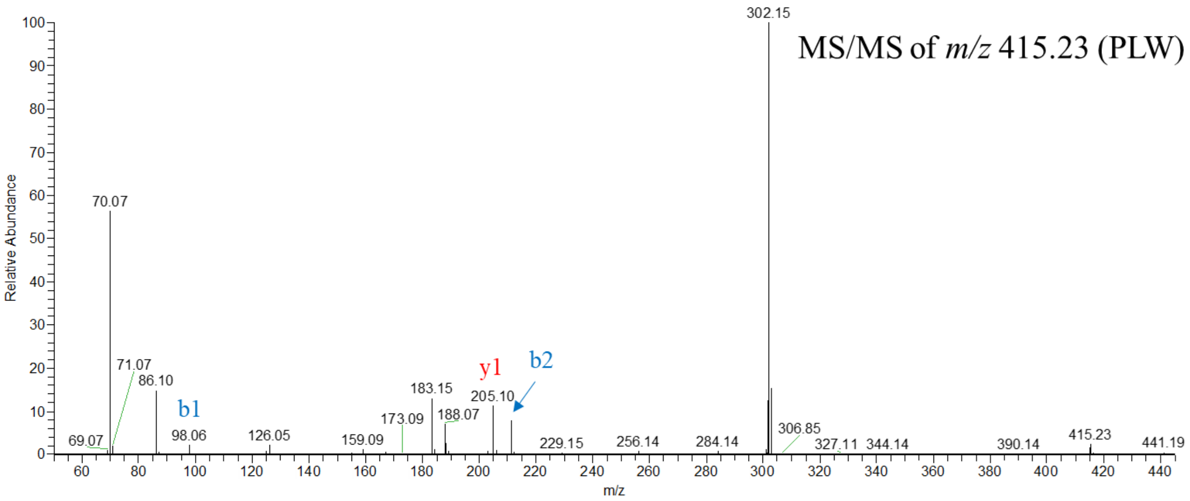
MS/MS of m/z 415.23 (PLW)
Relative Abundance
m/z
302.15
70.07
71.07
86.10
69.07
98.06
126.05
159.09
173.09
183.15
188.07
205.10
229.15
256.14
284.14
306.85
327.11
344.14
390.14
415.23
441.19
b1
y1
b2

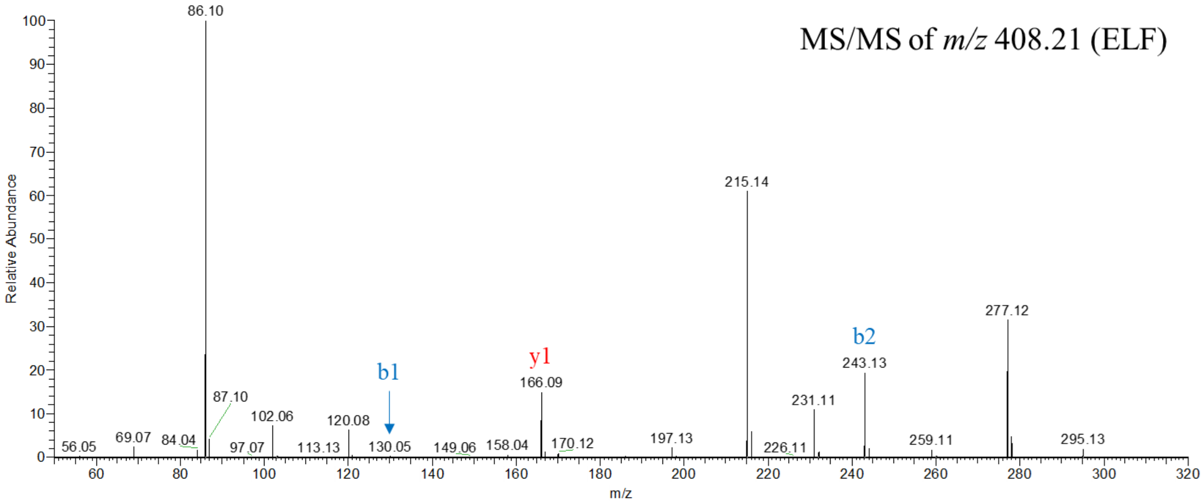
MS/MS of m/z 408.21 (ELF)
Relative Abundance
m/z
86.10
215.14
277.12
243.13
231.11
166.09
56.05
69.07
84.04
87.10
97.07
102.06
113.13
120.08
130.05
149.06
158.04
170.12
197.13
226.11
259.11
295.13
b1
y1
b2

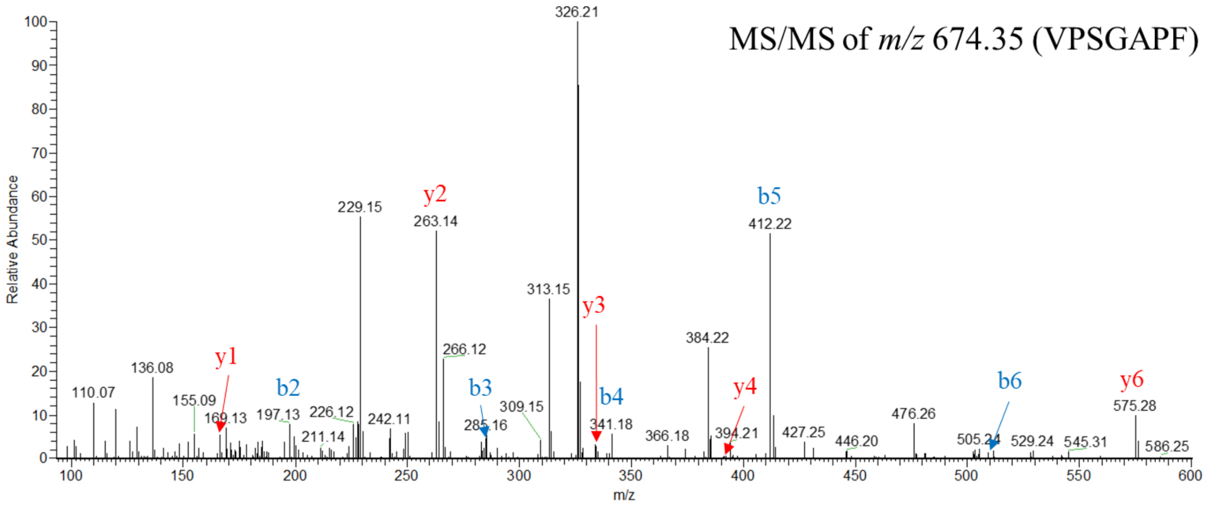
MS/MS of m/z 674.35 (VPSGAPF)
Relative Abundance
m/z
326.21
229.15
263.14
412.22
313.15
384.22
266.12
136.08
110.07
155.09
169.13
197.13
211.14
226.12
242.11
285.16
309.15
341.18
366.18
394.21
427.25
446.20
476.26
505.24
529.24
545.31
575.28
586.25
y1
b2
y2
b3
y3
b4
y4
b5
b6
y6

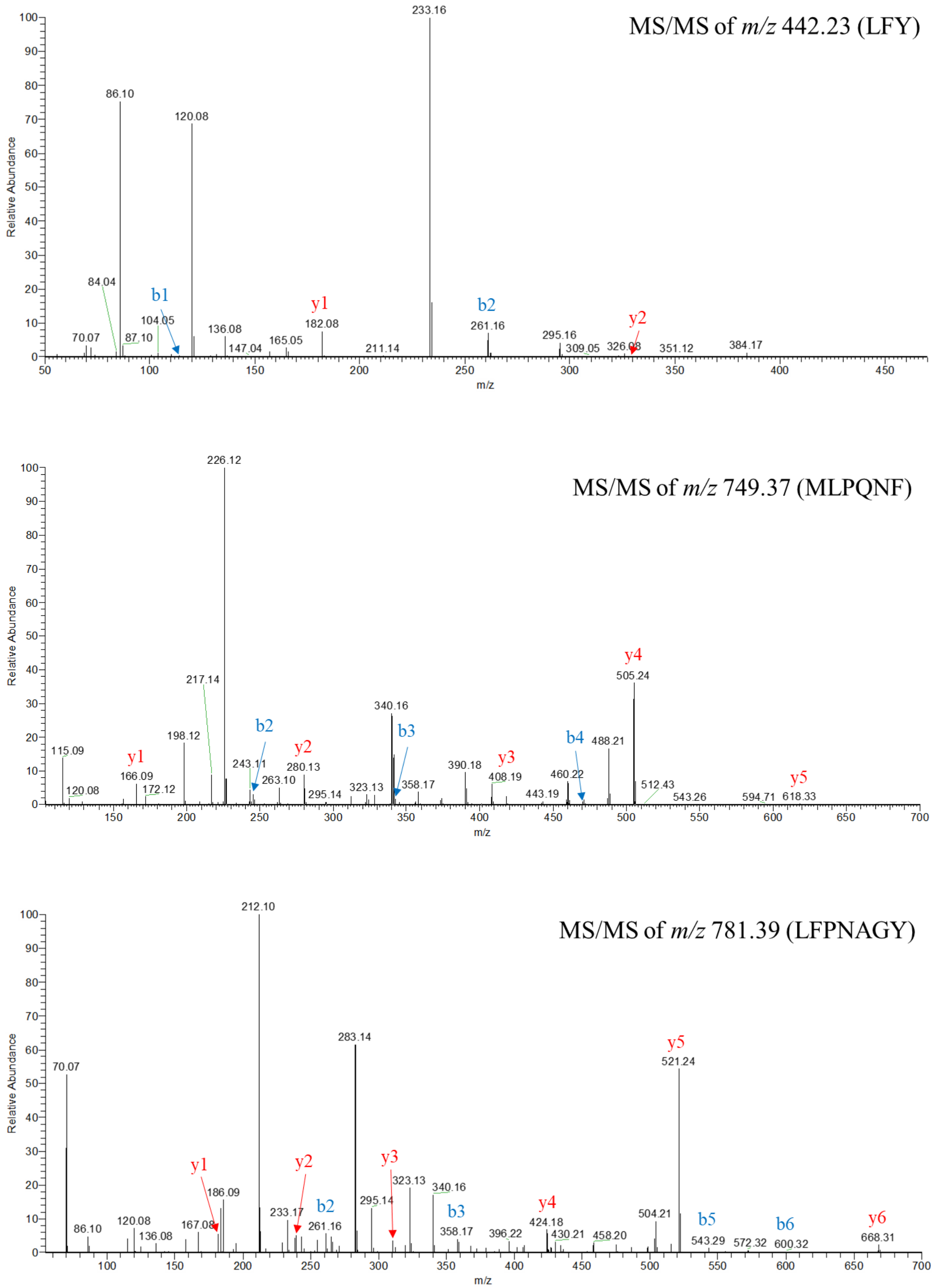

Figure S1. Manually confirmed nine peptides LC-MS/MS spectrum

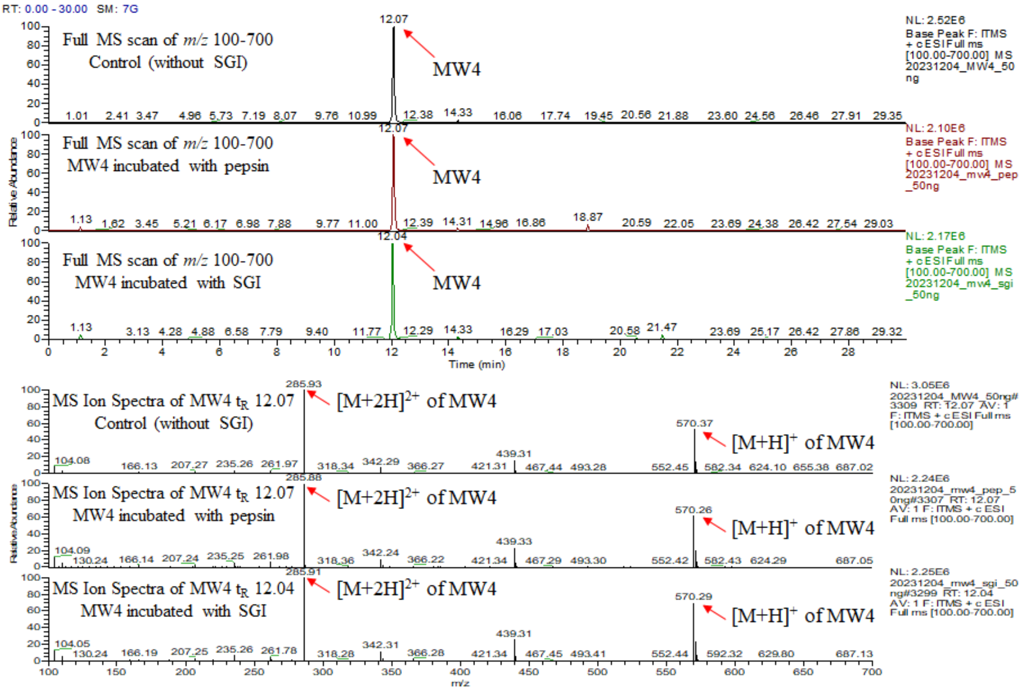

Figure S2. LC-MS chromatogram and MS spectra of the stability MW4 toward SGI digestion.

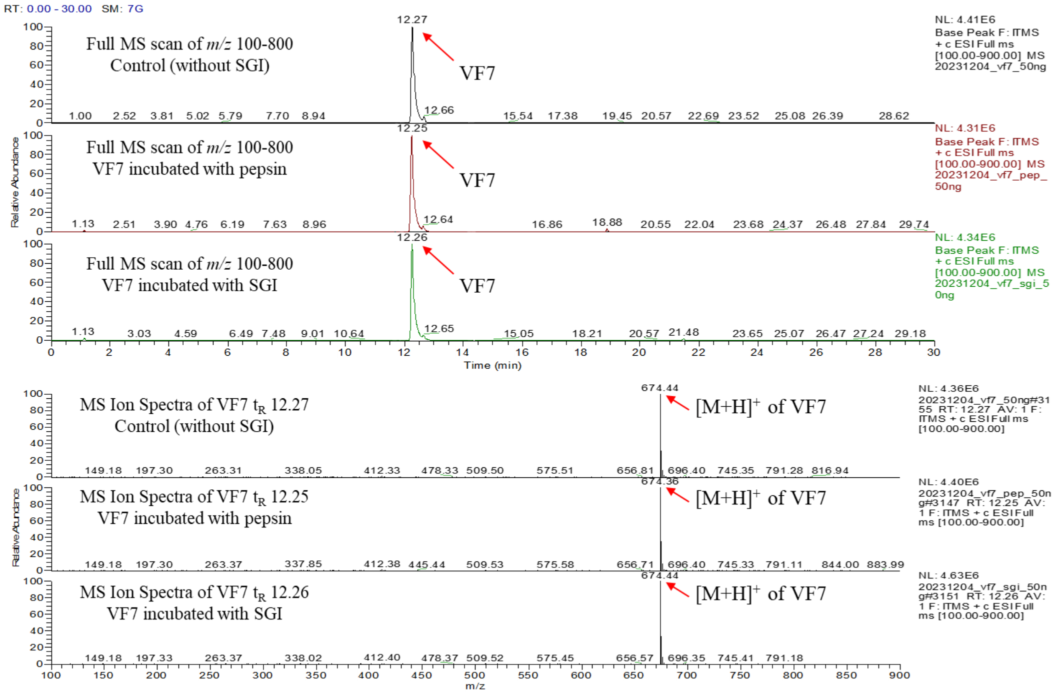

Figure S3. LC-MS chromatogram and MS spectra of the stability VF7 toward SGI digestion.

Table S1. Identified peptides from BMSP-GP F8

Identified Protein	Peptide Sequence	m/z (Observed)	Mass	Peptide Length
(gi|1229793389) sucrose-binding protein-like isoform X2 [*Momordica charantia*]	HGPGGDNPESF (HF11)	557.2332	1112.4523	11
	VPSGAPF (VF7)	674.3514	673.3435	7
	TSIPGQF (TF7)	749.3831	748.3755	7
(gi|1229785782) putative amidase C869.01 [*Momordica charantia*]	PGITVPAGY (PY9)	874.4664	873.4596	9
*de novo sequencing (only)	KQKTAWL (KL7)	437.7613	873.5072	7
	MPHW (MW4)	285.6278	569.2421	4
	HNLPVL (HL6)	692.4081	691.4017	6
	RATLPF (RF6)	352.7083	703.4017	6
	PLW (PW3)	415.2333	414.2267	3
	ELF (EF3)	408.2128	407.2056	3
	LFY (LY3)	442.2336	441.2263	3
	MLPQNF (MF6)	749.3650	748.3578	6
	LFPNAGY (LY7)	781.3879	780.3806	7
	SPPAL (SL5)	484.2769	483.2693	5
	TPPGSL (TL6)	571.3085	570.3013	6
	VMPHF (VF5)	315.6567	629.2996	5

*de novo sequencing (only): peptide absence in the protein database matching
